# Fermented Polyherbal Formulation Restored Ricinoleic Acid-Induced Diarrhea in Sprague Dawley Rats and Exhibited *In Vitro* Antibacterial Effect on Multiple Antibiotic-Resistant Gastrointestinal Pathogens

**DOI:** 10.1155/2024/1997064

**Published:** 2024-09-30

**Authors:** Subhanil Chakraborty, Babli Roy, Subhajit Sen, Santi M. Mandal, Arghya Das, Ranadhir Chakraborty

**Affiliations:** ^1^ Omics Laboratory, Department of Biotechnology University of North Bengal, Raja Rammohunpur, P.O. NBU, Darjeeling, West Bengal 734013, India; ^2^ Department of Chemistry Tufanganj Mahavidyalaya, P.O. Tufanganj, New Town, Coochbehar, West Bengal 736160, India; ^3^ DBT-NECAB Assam Agricultural University, Jorhat, Assam 785013, India; ^4^ Central Research Facility Indian Institute of Technology, Kharagpur, West Bengal 721302, India; ^5^ Department of Microbiology AIIMS, Madurai, Tamil Nadu 625002, India

## Abstract

The involvement of multiple antibiotic-resistant gastrointestinal pathogens in diarrhea aggravates the disease condition uncontrollably. The current study aimed to find and develop a suitable formulation utilizing multiple natural components from known plant sources to augment the current therapeutic outcomes. The hydroethanolic extraction method was applied through boiling and fermentation on ancient observation-based efficacious plant parts for developing the antidiarrheal polyherbal formulation AP-01. An animal study model of diarrhea was used to evaluate the safety and efficacy of the formulation. The formulation was tested *in vitro* on four different multiple antibiotic-resistant gastrointestinal pathogens collected from the national repository. The formulation depicted no cytotoxicity on normal gut cells and was efficacious at 10 ml/kg single dose in relieving symptoms of diarrhea by 79.71%, compared with the standard drug showing a reduction of symptoms by 83.01%. AP-01 exhibited delayed gastric motility. The symptoms of diarrhea ceased to occur within 320.66 ± 5.05 minutes with AP-01, whereas the standard drug took 308 ± 6.63 minutes. AP-01 was found successful at a viable dosage regimen of 75 *μ*l/ml v/v to 100 *μ*l/ml v/v in inhibiting the growth of different pathogens from the Enterobacteriaceae family possessing resistance against several classes of antibiotics in culture media. Chemical analysis revealed different alkaloids, flavonoids, and polyphenols that probably work in unison through multiple modes of action to arrest diarrhea and inhibit pathogens simultaneously. These promising results shown by AP-01 should evoke an effort to dive deep into research and development for better therapeutic formulations for infectious diarrhea by harvesting nature's arsenal.

## 1. Introduction

Gastrointestinal diseases, such as diarrhea, can easily overwhelm the public health system in developing countries. They spread quickly through food or drinking water and can lead to epidemics that cause significant loss of life even today.

Castor oil-induced diarrhea is often used in preclinical research to test drugs that control diarrhea because it provides a standardized and controllable method to induce diarrhea in laboratory animals, typically rodents [1, 2]. Castor oil contains ricinoleic acid that irritates the intestines and stimulates bowel movements, leading to diarrhea [3].

Infectious diarrhea, mainly due to pathogenic bacteria, is a significant public health problem worldwide. Major enteric bacterial pathogens responsible for causing diarrhea among human infants and young animals include several species of genera such as *Escherichia, Vibrio, Shigella, Salmonella, Campylobacter, Arcobacter, Yersinia, and Listeria*. Even within the genus *Escherichia, diarrheagenic Escherichia coli* (DEC) pathotypes, particularly *enteropathogenic E. coli* (EPEC), *enterohemorrhagic E. coli* (EHEC), *enterotoxigenic E. coli* (ETEC), *enteroaggregative E. coli* (EAEC), and *enteroinvasive E. coli* (EIEC), are frequently associated with diarrheal cases [4, 5]. Pathogens adhere to the intestinal epithelial cells using adhesins, which are specialized proteins that bind to the host's cell receptors. Once attached, these bacteria colonize the gastrointestinal lining and may form biofilms, which protect them from the host immune system and antibiotics. They can evade the immune system by modifying surface antigens and secreting factors that inhibit immune responses. Gastrointestinal pathogens release toxins that damage host cells and the mucosal barrier and disrupt gastric functions. Infection leads to an inflammatory response that can result in gastritis, ulcers, and even gastric cancer over time [6].

In developing nations, including India, there is a significant hindrance to the availability of all the standard cultures for diagnosis and proper medication to treat the ailment. Even though economic development and progress in healthcare delivery are expected to catalyze substantial improvements in infectious disease-related morbidity and mortality by the year 2020, it is predicted that diarrhea will remain a leading health problem. The main cause of death from diarrhea is dehydration, which results from the loss of electrolytes in diarrheal stools [7]. During the past decade, there have been some significant improvements in treating infectious diarrhea. Oral rehydration therapy has contributed greatly to the reduction of diarrheal mortality rates in children and older people. However, the diarrheal attack rate has remained unchanged, and this treatment often fails in the high stool output state [8]. Symptomatic therapy with antimotility agents is restricted to nondehydrated patients without features of systemic infection. These are not indicated in infants and patients with febrile bloody diarrhea. Moreover, there is an increasing threat of drug resistance, side effects of antibiotic treatment, and suprainfection when normal flora is eradicated with antimicrobial agents. Vaccines have been considered the most feasible approach to diarrheal management. Various attempts have been made to develop vaccines against diarrhea-causing organisms [8–10]. However, the response to such vaccines in developing countries, particularly for toddlers, has not been encouraging [11]. Considerable technical barriers need to be overcome before continuing clinical evaluation of prospective vaccine candidates. Thus, an important niche exists for developing cost-effective alternative approaches, and medicinal plants may serve to fulfil this niche.

Various medicinal plant preparations have been identified for treating diarrhea, but their mechanism for eliminating organisms that cause diarrheal diseases needs to be better comprehended. While data are available on the effect of different plants on intestinal motility in experimental models and antibacterial action [12, 13], there is a lack of information on the efficacy of more than one medicinal plant working synergistically as a polyherbal formulation on various aspects of diarrheal pathogenicity [14]. The current study aims to find a suitable arsenal from nature with the help of ancient knowledge of Ayurveda to introspect into the safety and efficacy of such preparation through in vivo and in vitro studies and to shed some light on the chemical constituents attributed to such actions. Diarrhea associated with gastrointestinal infections caused by multiple antibiotic-resistant bacteria could constitute a diverse series of problems involving the gut health in general [15]. Integrating evidence-based traditional knowledge of safe and efficacious medicinal plants and nature-based drug discovery, using common plant parts, may contribute to the development of a sustainable, safe, and cost-effective polyherbal therapeutic formulation that can pose a threat to the multiple antibiotic-resistant pathogens yet producing restorative actions on gut microbes [16, 17]. Innovation in drug discovery often relies upon advancements of research in complimentary and natural medicine for unencumbering the complexity and cost of drug manufacturing, where the therapeutic preparation could be made with readily available materials using simple apparatus and methodology [18].

The present study tries to explore the possibility of management of infectious diarrhea, where a single polyherbal therapeutic preparation could be used to fight against both the menace of secretary diarrhea and the infection caused by gastrointestinal pathogens, which is not yet available or indicated for therapeutic use. The findings from this study could be used to reveal the mode of action of such potential preparation in future detailed experiments.

## 2. Materials and Methods

### 2.1. Plant Parts for Preparing the Fermented Polyherbal Formulation

Fermented polyherbal formulation (AP-01) was prepared in the laboratory by boiling dried and cleaned stem barks of *Holarrhena antidysenterica* and *Gmelina arborea* mixed with large-sized dry resins of *Vitis vinifera* in the presence of dried flowers of *Madhuca indica* in potable drinking water. Dried flowers of *Woodfordia fruticosa* were added after the water volume lowered to one-quarter of the initial volume for initiation of fermentation while using jaggery as a source of sugar. After fermentation was completed in about 45 days, the formulation was duly filtered and kept in sterile dark glass bottles after passing through bacterial filters of 0.2-micron pore size for further use. The preparation method was modified from the Ayurvedic Pharmacopoeia of India following Sharangadhara Samhita and Vaisajyaratnavali references for different “Arishtha” formulations. All plant parts were procured from the local market and identified by qualified Ayurveda practitioners with formal academic degrees for practicing Ayurveda [19–21].

Loperamide hydrochloride (Imodium brand of Johnson and Johnson Consumer Inc.) was used as a standard drug against diarrhea induced by the effects of castor oil.

Castor oil (Erand Tail brand of Dabur) was used to induce diarrhea in animals.

Ultrapure kaolin from HiMedia Laboratories was used as a marker for determining the residual effect of AP-01 on the gastric motility of animals.

### 2.2. Experimental Animals

36 Sprague Dawley rats of either sex, aged around six weeks, weighing 80 to 120 grams, were obtained from the animal house of the Department of Zoology, University of North Bengal. Animals were acclimatized for seven days under standard environmental conditions, and a pellet diet of chow and water was provided in copious amounts. Experiments were conducted after the animals were deprived of both food and water for 18 to 20 hours. The study followed the National Research Council's Guide for the Care and Use of Laboratory Animals and was approved by the Institutional Animal Ethics Committee of the University of North Bengal, Siliguri District, Darjeeling, West Bengal, India (Ref. No. IAEC/NBU/2018/04 dated 12.09.2018) [22].

### 2.3. Microorganisms with Pathogenesis and Nonpathogenic Properties

Different human pathogenic bacterial clinical isolates were thankfully received from the Gastrointestinal Tract Pathogens Repository (GTPR) of the National Institute of Cholera and Enteric Diseases (NICED), sponsored by the Indian Council of Medical Research (ICMR), the apex body to conduct clinical and preclinical research in India. Based on the availability at the repository and varying degrees of resistance against multiple antibiotics, four different strains were chosen for performing in vitro tests with AP-01. All four strains were received in sealed vials with extensive care and caution, and further experiments were conducted aseptically in the biosafety level II laboratory (Omics Laboratory) as per the guidance of ICMR. Proper disposal by autoclaving all media containing GTPR strains and thereafter incinerating the loads of the autoclave were followed. *Escherichia coli* 01241 (identification number assigned to each clinical isolate by GTPR) resistant to ceftazidime (CAZ), aztreonam (AT), ceftriaxone (CTR), trimethoprim/sulphamethoxazole (SXT), nalidixic acid (NA), ampicillin (AMP), and cefotaxime (CTX); *Salmonella* Typhi 01396 enterica serovar resistant to streptomycin (S), amoxiclav (AMC), ciprofloxacin (CIP), norfloxacin (NOR), doxycycline (DO), and erythromycin (E); *Salmonella* Typhi 01263 enterica serovar resistant to ofloxacin (OFX), ciprofloxacin (CIP), nalidixic acid (NA), and norfloxacin (NOR); and *Shigella boydii* 01399 strain resistant to nalidixic acid (NA), ciprofloxacin (CIP), cefotaxime (CTX), and kanamycin (K) were received from GTPR [23]. All four GTPR strains, along with a laboratory isolate of a nonpathogenic nature, *Escherichia coli* K12, were treated with the polyherbal formulation AP-01 to conduct further research after determining the susceptibility/resistance pattern of the microorganisms.

Antibiotic resistance/susceptibility profiles of the above-mentioned bacterial strains were also determined at the lab as per the guidelines of CLSI (Clinical Laboratory Standards Institute) (CLSI, 2022) using the Kirby–Bauer disk diffusion method. The laboratory findings mostly corroborated with the resistance details available with all GTPR strains. The zone of inhibition was measured and compared with breakpoints given by EUCAST/CLSI to determine the resistance/susceptibility profile of each strain against antibiotics belonging to different classes [24, 25].

### 2.4. Phytochemical Screening of the Formulation

Primary phytochemical studies were performed following the standard biochemical qualitative and analytical assay procedures and literature surveys for individual plant material and the polyherbal formulation to confirm the presence of phytoconstituents [26–28]. Finally, the polyherbal formulation was characterized by different spectroscopic techniques such as FTIR spectroscopy (Nicolet 6700 FTIR spectrometer, Thermo Fisher Scientific, USA), ^1^H NMR spectroscopy (600 MHz, Bruker), and high-performance liquid chromatography-tandem mass spectrometry (HPLC-MS/MS, Waters, USA, HPLC-MS system equipped with model 2525 pump, ZQ detector) with acetonitrile and water (95 : 5) using conessine as the biomarker. Briefly, the alkaloid fraction was subjected to HPLC-MS using the solvent system of acetonitrile:water (both containing 0.1% acetic acid) at a flow rate of 1 ml/min [28].

### 2.5. Antimicrobial Assay of Polyherbal AP-01 against GTPR (GTPR Signifies Gastrointestinal Tract Pathogens Repository of Indian Council of Medical Research, Govt. of India) Pathogenic Strains

The minimum inhibitory concentration (MIC) of polyherbal preparation AP-01 against the four GTPR strains and *Escherichia coli* K12 was determined by the serial broth dilution method by adhering to the Clinical Laboratory Standards Institute (CLSI, 2022) reference method [24]. Polyherbal preparation AP-01 was added to sterile 5 ml Mueller–Hinton broth in different concentrations from 10 *µ*l/ml to 200 *µ*l/ml with a gradual increment of 5 *µ*l/ml, and then, 1% aliquot of the mid-log-phase culture of 0.5 McFarland standard of each bacterium was inoculated in every tube equally and incubated for 24 h at 37°C. After incubation, growth was measured in a spectrophotometer (SPECTROstar Nano—BMG LABTECH) at 600 nm to determine the minimum inhibitory concentration (MIC). All the experiments were performed in triplicate and repeated twice. The overnight-grown mid-log-phase bacterial culture was inoculated both in the presence of polyherbal AP-01 at the MIC dose and without the presence of polyherbal AP-01 at 37°C and incubated for six hours to compare the optical density-based growth under both conditions using the spectrophotometer. At the same time, serially diluted aliquots from cultures grown under both conditions (with and without adding AP-01) were grown over Luria agar plates through the spread plate technique for reassurance of growth/inhibition of growth by counting the colony-forming units (cfu) of the strains. The highest dilution spread over the Luria agar plates, which showed no growth, was the minimum bactericidal concentration (MBC). In the laboratory, *Escherichia coli* K12 was isolated and grown in Mueller–Hinton Broth (HiMedia) with and without AP-01. This experiment was conducted to study the inhibitory pattern and determine the MIC and MBC values of AP-01 against the strains.

Furthermore, the modified Mueller–Hinton agar well diffusion method was also performed to determine the zone of inhibition produced by different volumes of undiluted AP-01 against each bacterial strain compared with the negative control (DMSO (DMSO is dimethyl sulphoxide)) and positive control (meropenem 10 *μ*g). 100 *μ*l aliquot of mid-log-phase culture of 0.5 McFarland standard containing 1 × 10^9^ cfu/ml of each bacterium was aseptically spread over 20 ml Mueller–Hinton agar plates, and sterile 6-mm well borers were used to dig wells. Serially diluted volumes of 150 *µ*l, 125 *µ*l, 100 *µ*l, 75 *µ*l, 50 *µ*l, and 25 *µ*l AP-01 in DMSO (volume adjusted to 150 *μ*l) and 150 *μ*l DMSO were poured into individual wells, and meropenem disks were placed in separate plates for each strain and incubated at 37°C for 24 hours to determine the zones of inhibition. Tests were performed in triplicate.

### 2.6. Analysis of Swarming Motility of the GTPR Pathogen Salmonella Typhi Serovar 01263 Enterica in the Presence and Absence of AP-01

A swarm agar [0.7% (W/V) Bacto-agar, 0.5% (W/V) glucose] plate was used to observe *Salmonella* swarming motility [29, 30]. Gentle spotting of 2 *µ*l of fresh *Salmonell*a culture (OD_600_ value of 0.4) was performed on the surface of the swarm agar and in the center. After 8 h of incubation at 30 °C, the observed growth of the migrated cells around the inoculation site was photographed. A sub-MIC dosage of AP-01 was added to the swarm agar medium to demonstrate the inhibition of swarming motility of the same *Salmonella* strain following the same protocol.

### 2.7. Primary Acute Oral Tolerance Tests

After acclimatization, animals were distributed into several groups to check tolerance imparted by AP-01 when fed through the oral route. The standard dichromate oxidation method was applied to determine the level of ethanol in AP-01 formulation, and it was determined to be present at a quantity less than 3% v/v at the time of study (data not given). A dosage of up to 50 ml/kg for the body weight of rats was found to be well tolerated and not producing any symptoms of acute toxicity or lethality. During the study of evaluating oral acute toxicity testing, no mortality, changes in the general behavior or agility, alterations in the food intake or water intake, or abnormal changes in body weight were observed. Further studies were carried out using 5 ml/kg and 10 ml/kg dosing of AP-01 as rationalized in some earlier studies using plant extracts [31, 32] and according to the Organization of Economic Corporation Development Guidelines [33] following the principles and criteria summarized in the Humane Endpoints Guidance Document. The rationale for such dosing was based on the body surface area ratio for extrapolating human dose to animals.

### 2.8. Cytotoxicity Study

The human intestinal epithelial cell line IEC 18 and rat intestinal epithelial cell line RIE-1 were, respectively, procured from the National Centre for Cell Science, Pune, and the Centre for Cell and Molecular Biology, Hyderabad, India, for performing the MTT assay with different strengths of AP-01 and by diluting AP-01 with DMSO. Dulbecco's modified Eagle medium, fortified with 0.25% sodium bicarbonate solution and 8% fetal bovine serum, was used during the maintenance of the cell culture. The cell lines were stored at 37°C in a 5% CO_2_ atmosphere in a CO_2_ incubator (Thermo Fischer Scientific) with 90% humidity. An equal number of cells were placed at 96-well plates with 100 *μ*l culture media. After 24 hours of incubation, the media were replaced by 25 *μ*l of 18.75 *μ*l/ml, 37.5 *μ*l/ml, 75 *μ*l/ml, 150 *μ*l/ml, and 750 *μ*l/ml AP-01 after adjusting the volume to 100 *μ*l by adding DMSO and another well with 100 *μ*l DMSO as a control. After incubation under a similar storage condition for 24 hours, the entire contents of each well were replaced by 10 *μ*l of 5 mg/ml MTT solution freshly prepared in phosphate buffer saline and incubated for another 6 hours. Succinate dehydrogenase enzyme present in cell lysates of viable cells would therefore be reacting with yellow MTT solution (3-(4,5-dimethylthiazol-2-yl)-2,5-diphenyl-2H-tetrazolium bromide) to form a violet-blue crystalline formazan molecule that could be dissolved in propan-3-ol or isopropyl alcohol for visible spectroscopy for quantification. As metabolically active cells could only produce formazan crystals, their quantification was used to determine the number of living cells. The maximum amount of formazan should be formed in the well containing DMSO as a control. The plates were horizontally shaken after adding 50 *μ*l of isopropyl alcohol for 2–3 mins. Absorbance was taken at *λ*_max_ of 600 nm, concerning the violet-blue color of formazan at 490 nm, by a 96-well plate reader (CLARIOstar Plus, BMG Labtech). The percentage of cytotoxicity was calculated by comparing the OD value of AP-01-treated cells with the control, (OD_Control_–OD_Treatment_)/OD_Control_ × 100.

AP-01 was added to the cell lines as 0.25–MIC, 0.5–MIC, MIC, 2X MIC, and 10X MIC dosage towards lab isolate bacterial strain *E. coli* K12 [34, 35].

Human small intestine-derived cell line HIEC 6 from ATCC was further used to determine the broad-spectrum of noncytotoxicity produced by AP-01.

### 2.9. Experimental Design for the Action of AP-01 on the Symptom of Diarrhea Induced by Ricinoleic Acid from Castor Oil

The animals were divided into six groups, each containing six randomly selected but an equal number of male and female Sprague Dawley rats for a parallel experimental design to study the effect of AP-01 on castor oil-induced diarrhea. Pregnancy, prior illness, and underweight (<100gm) were considered the exclusion criteria of the animals. The animals were randomized and included in each group using standard randomization prompts using MS Excel software. The normal group received only distilled water (10 ml/kg p.o.), and the negative control group received only castor oil at a dose of 10 ml/kg. The positive control group was fed a per oral dose of 2 mg/kg loperamide hydrochloride suspended in distilled water half an hour before receiving castor oil (10 ml/kg *per oral*). The first two test groups consecutively received 5 ml/kg and 10 ml/kg AP-01 half an hour before receiving castor oil (10 ml/kg) through the oral route. However, the last test group received AP-01 at a dose of 10 ml/kg half an hour after feeding castor oil at a dose mentioned previously [31, 32]. Each animal was observed under a glass funnel lined with blotting paper for twenty-four hours after feeding castor oil and water in the case of the standard group. Observations were made to test the parameters such as the onset of symptoms of diarrhea employing excreting wet (watery/diarrheagenic) feces, cessations of diarrhea using the end time for the expulsion of wet feces, the persistence of symptoms of diarrhea, the total weight of feces, weight of wet feces, total number of stool pellets, and the total number of wet stool pellets. The percentage of inhibition of symptoms of diarrhea was determined by comparing the decrease in the persistence of diarrhea symptoms, mass of wet fecal material, and number of wet stools expelled during the observation period by animals of the positive control group and test groups to the negative control group animals. As the study did not involve any invasive procedures, the same animals were given 5–7 days of resting period before the next gastric motility-related experiments. All the test animals were rehabilitated and rehoused for further use.

### 2.10. The Residual Effect of AP on Body Weight, Food and Water Intake, and Gastric Motility (Using Kaolin as a Marker) before and after Intervention

The animals were again divided into six groups, each group containing six randomly selected but comprising an equal number of male and female Sprague Dawley rats for a parallel experimental design to study the effect of AP-01 on body weight, food and water intake, and gastric motility rates using kaolin as a marker. Each animal was weighed one day before experimenting and again weighed after 18–20 hours from the conclusion of the observation time of the investigation. Food and water intake was calculated one day before intervention by providing a fixed amount of pellet diet and a fixed volume of water and calculating the leftover mass of food and volume of water after twenty-four hours. Dietary intake and water intake were again measured after twenty-four hours from the interim time of twelve hours of observation of the experiment as all animals were fed with a fixed amount of diet and water after twelve hours of observation.

Before feeding the animals after the observations, each animal was administered with 2 ml/kg *per oral* aqueous solution of kaolin. As identified from previous studies, a dose of 40% aqueous solution of kaolin could be used as a marker of gastric motility rate as it could alter the color of stool pellets without affecting the drug action administered previously. Therefore, the measured time gap between the feeding of kaolin and expulsion of fecal matter marked with kaolin for each animal could serve as a determinant of gastric motility because it could be assessed as the time taken by the kaolin to get a passage through the gastrointestinal tract of the rats. Longer latency before the expulsion of kaolin-marked fecal matter could suggest decreasing gastric motility rates [32, 36].

### 2.11. Statistical Analysis

The observational and all measured values were reported as mean ± standard error of mean (S.E.M.) or mean ± standard deviation (SD). Percentage changes compared to normal values and control group data were carried out as and when required. Paired and unpaired “Student's *t*-test” was employed for statistical analysis for the validation of inference at a level of significance of *P* < 0.001, which was considered statistically significant.

## 3. Results

### 3.1. Presence of Probable Phytochemicals

**Figure 1:**
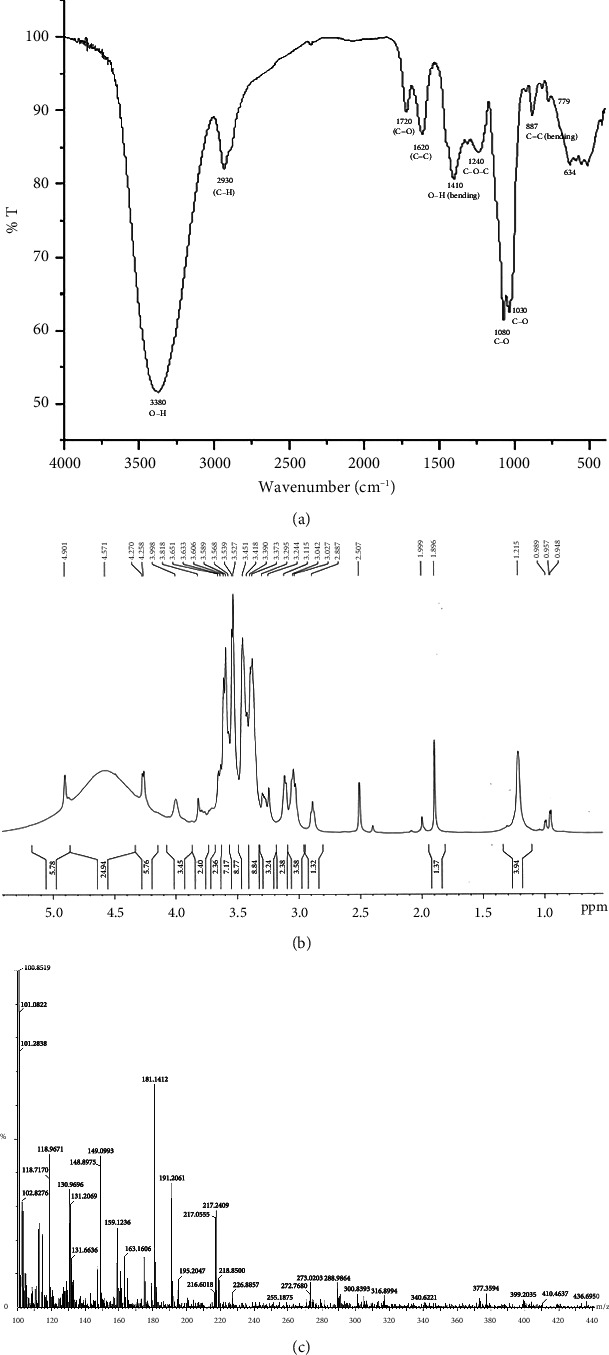
(a) FTIR, (b) NMR, and (c) LC-MS study of fermented polyherbal formulation AP-01.

The presence of different functional groups, viz., hydroxyl (alcoholic and phenolic-OH), carbonyl (C=O), alkoxy(C-O), epoxy (C-O-C), C-H, C=C, and C-H bonds was confirmed by the Fourier transform infrared (FTIR) spectra of our polyherbal formulation performed in liquid form (Figure 1(a)) [37]. The high intense peak at 3380 cm^−1^ confirms the predominant presence of hydroxyl groups (alcoholic/phenolic) in the present formulation. This also indicates the considerable existence of alcohols and polyphenols in our formulation, which could be responsible for their significant pharmacological activity. Besides, the ^1^H-NMR spectra of our current polyherbal formulation further indicate the presence of primarily aliphatic protons (*δ*; 1.25–4.9 ppm) and alcoholic/phenolic/benzylic protons (chemical shift values, *δ* 4.26, 4.57, and 4.9 ppm (Figure 1(b)).

On account of the FTIR and 1H-NMR spectra along with the LC-MS/MS data (Figure 1(c)), the presence of diverse phytochemicals, viz., quercetin(MW 302.23), *β*-sitosterol (MW 414.7), n-octacosanol (MW-395.72), kurchine (MW-342.6), conessine (MW 356.58), antidysentericine (MW-356.5), conimine (MW-328.53), gmelinol (MW-402.4), luteolin (MW-286.24), gmelanone (368.33), stigmasterol (MW-412.69), betulinic acid (MW-456.700), myricetin (MW-318.235), catechin (290.2681), kaempferol (MW-286.236), beta-Amyrin (MW-426.717), luteolin (MW-286.23), n-hexacosanol (MW-382.70), oleanic acid (MW-456.7), linolenic acid (MW-278.42), mannitol (MW-182.17), myricetin (MW-318.23), and their various glycosidic derivatives, is primarily inferred as bioactive compounds. The chemical composition was also compared with the literature on individual plant extracts. Hence, the major classes of phytochemicals present in our polyherbal formulation are alkaloids, flavonoids, and polyphenols.

### 3.2. Cytotoxicity Analysis

AP-01 was not found to be producing any cytotoxic effect in either concentration as 0.25—MIC, 0.5—MIC, MIC, 2X MIC, and 10X MIC dosages to either human or rat intestinal epithelial cell lines as at the highest concentration; cell viability was not found to be reduced for more than 10 percent as represented in Table 1.

Data are presented as mean ± SD from three independent experiments, each run in triplicate. The percentage inhibition was calculated as the percent difference between growth in DMSO and growth in the presence of AP-01 after 24 h of incubation.

### 3.3. Effect of AP-01 on Animals with Diarrhea Induced by Castor Oil

**Figure 2:**
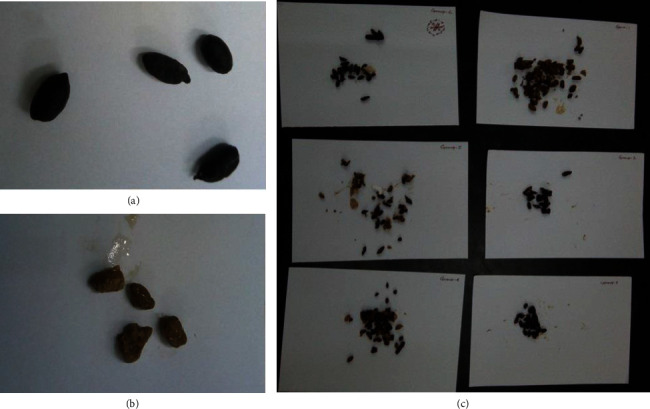
(Colour) (a) Normal stool, (b) wet stool, and (c) comparative normal and wet fecal materials of all animal groups starting clockwise from the top right corner to the top left corner, where the top right corner depicted the fecal material of the normal group.

**Figure 3:**
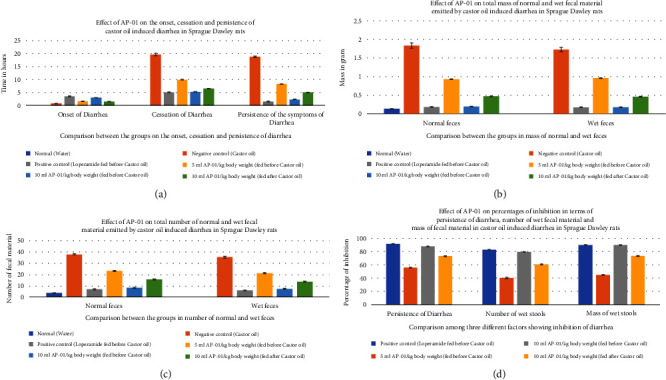
Effect of AP-01 on castor oil (0.8 ml)-induced diarrhea in Sprague Dawley rats. An average of six determinations is represented in each value expressing mean ± standard error of the mean. *P* < 0.001 vs. control, Student's *t*-test. (a) Effect of AP-01 on the onset, cessation, and persistence of castor oil-induced diarrhea in Sprague Dawley rats. (b) Effect of AP-01 on the total mass of normal and wet fecal materials emitted by castor oil-induced diarrhea in Sprague Dawley rats. (c) Effect of AP-01 on the total number of normal and wet fecal materials emitted by castor oil-induced diarrhea in Sprague Dawley rats. (d) Effect of AP-01 on percentages of inhibition in terms of persistence of diarrhea, number of wet fecal materials, and mass of the fecal material in castor oil-induced diarrhea in Sprague Dawley rats.

All fecal excrements were collected, weighed, and analyzed for the study (Figure 2). During the period of observation for twenty-four hours after dosing with castor oil, all animals of the negative control group showed symptoms of diarrhea with an early onset of the release of a wet fecal pellet within an hour (an average of 52 minutes) and the symptoms persisted as long as nineteen hours. Animals belonging to the first and second test groups showed dose-dependent delayed onset of symptoms; however, the occurrence ceased at a significantly earlier time (three hours) in the case of rats pretreated with 10 ml/kg AP-01 formulation. The third test group, which consisted of animals that received AP-01 after half an hour of castor oil feeding, also showed a delayed onset of symptoms compared to the negative control and first test groups. Treatment with loperamide as a standard drug produced a significant reduction of symptoms at a rate of 91.77, 83.1, and 89.82 in terms of the persistence of diarrhea, number of wet stools, and mass of wet stools, respectively, while the test groups having 5 ml/kg and 10 ml/kg AP-01 dosages as pretreatment and 10 ml/kg AP-01 as posttreatment with castor oil showed a consecutively reduced persistence of diarrhea at a percentage inhibition of 55.89%, 87.65%, and 73.21%, as shown in Figure 3. The test group that received 10 ml AP-01/kg body weight also showed a percentage of inhibition at 79.71% and 89.66% in terms of the number and mass of the wet fecal material considering the values of the negative control group as 100%. The duration of diarrhea was found to be significantly decreased in the case of the positive control group and second test group (that received 10 ml/kg AP-01 pretreatment) and measured consecutively as 94 minutes and 140 minutes, respectively. The normal animal group did not show any symptoms of diarrhea throughout the experiment and observational period.

### 3.4. The Residual Effect of AP-01 on Body Weight, Food and Water Intake, and Gastric Motility

**Figure 4:**
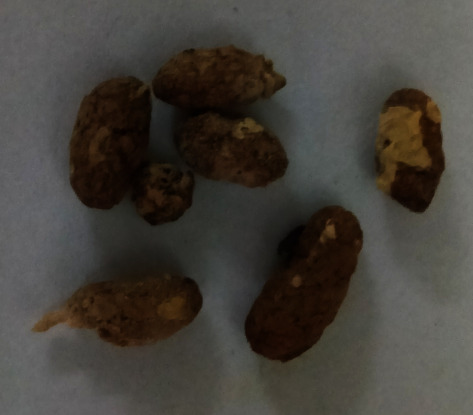
(Colour) Kaolin expulsion through normal feces as a marker of gastric motility.

**Figure 5:**
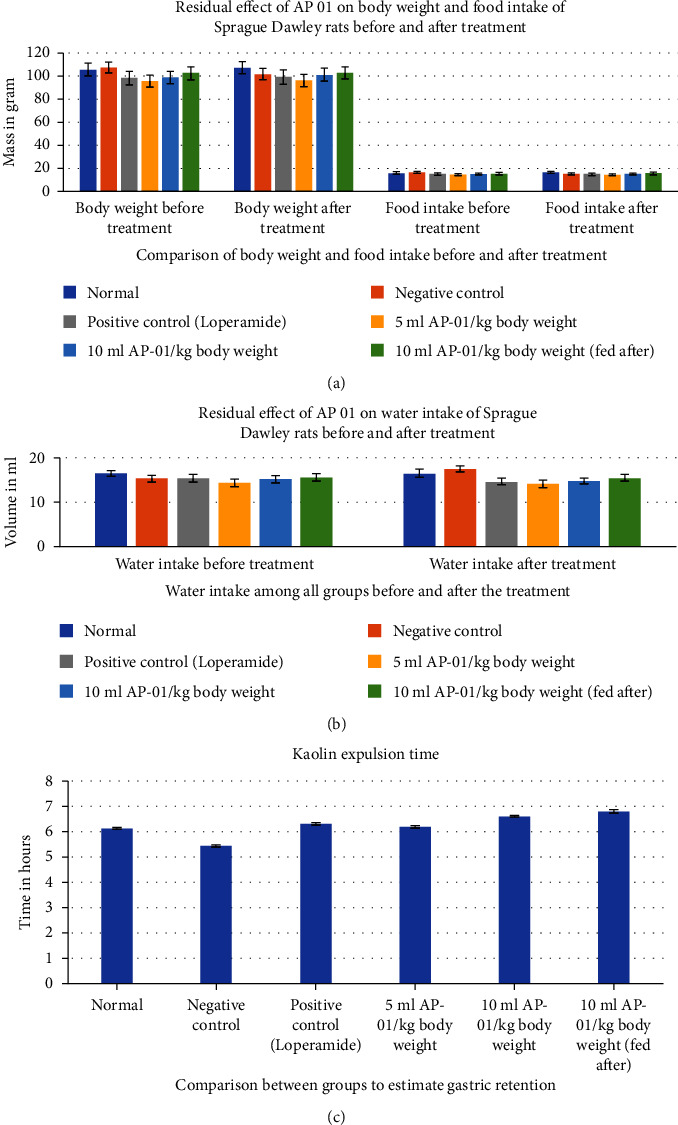
The residual effect of AP-01 on body weight, food and water intake, and gastric motility of Sprague Dawley rats. An average of six determinations is represented in each value expressing mean ± standard error of the mean. *P* < 0.001 vs. control, Student's *t*-test. (a) Residual effect of AP-01 on the body weight and food intake of Sprague Dawley rats before and after treatment. (b) Residual effect of AP-01 on the water intake of Sprague Dawley rats before and after treatment. (c) Kaolin expulsion time representing gastric motility.

Expulsions of stool pellets marked with kaolin were visually altered in color (Figure 4). Kaolin-marked stool pellets took a significantly longer time to pass through the gastrointestinal tract of animals in the case of both the groups treated with 10 ml/kg AP-01. However, a considerably higher latency period was shown when the dose of AP-01 was given at a later period (in the third test group). Body weight and food intake were reduced significantly in the case of the negative control group receiving only castor oil; however, there were no significant changes in the body weight or food intake for the animals belonging to any test groups and the positive control group. There was a substantial increase in the food intake for the animals of the normal group. Another significant change is shown in Figure 5, depicting the increase in the water intake only for the animals in the negative control group.

### 3.5. The Effect of AP-01 on Gastrointestinal Pathogens Collected from GastroIntestinal Tract Pathogens Repository

*E. coli* 01241, *S.* Typhi 01263, *S.* Typhi 01396, and *Shigella boydii* 1399 strains of pathogenic bacteria were collected from NICED to conduct in vitro antimicrobial tests on human pathogenic bacteria with AP-01. Primary tests confirmed the inhibitory effect. AP-01 was found efficacious against NICED-GTPR multiple antibiotic-resistant human pathogenic strain *E. coli* 01241 (resistant to CAZ, AT, CTR, SXT, NA, AMP, and CTX); *S.* Typhi 01396 (resistant to S, AMC, CIP, NOR, DO, and E); *S.* Typhi 01263 (resistant to OFX, CIP, NA, and NOR); and *Shigella boydii 1399* (resistant to NA, CIP, CTX, and K) (Table 2). The minimum inhibitory concentrations of AP-01 against each GTPR strain, determined through the broth dilution method and well diffusion method, were 80 *μ*l/ml v/v for *E. coli* 01241 and 100 *μ*l/ml v/v for the other three strains. The MIC dose of AP-01 for the lab isolate *E. coli* K12 was determined to be 75 *μ*l/ml v/v (Tables 3 and 4).

### 3.6. Analysis of Swarming Motility of the GTPR Pathogen Salmonella Typhi Serovar 01263 Enterica in the Presence and Absence of AP-01

**Figure 6:**
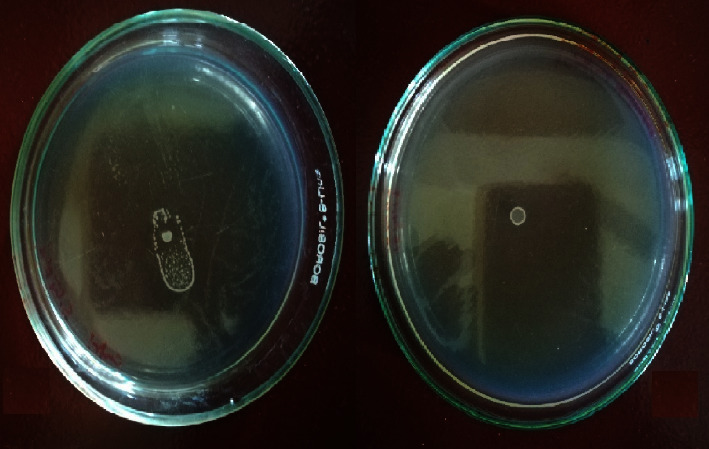
(Colour) Inhibition of swarming motility of *Salmonella* Typhi serovar 01263 on swarm agar supplemented with AP-01.

The presence of a sub-MIC dose of AP-01 (50 *μ*l/ml v/v) in semisolid media significantly arrested the swarming motility of the GTPR strain *S.* Typhi serovar 01263, the only strain to show swarming motility. In Figure 6, the first left side figure shows the swarming motility on the swarm agar plate observed without the presence of AP-01 in the media, and the adjoining figure shows the inhibition of swarming motility in the presence of the sub-MIC dose of AP-01 in media.

### 3.7. Statistical Analysis

Standard deviation and standard error of mean were determined using Microsoft Excel 2013 software. We used the *t-*test calculator (graphpad.com/quickcalcs/ttest1.cfm? Format = SD) for conducting both paired and unpaired Student's “*t*-tests.” A two-sample *t*-test is used to compare two datasets to see if their means are statistically different. In the case of onset and cessation of diarrhea, the results of the positive control group and three test groups were found to be significantly different compared to the negative control group, whereas there were no significant differences found between the results of the positive control group fed with loperamide as a standard drug and the second test group that received 10 ml AP-01/kg body weight half an hour prior to ingestion of castor oil in paired *t*-test (*P* value <0.001). An unpaired “*t*-test” was performed for the statistical analysis of the mass of normal and wet fecal material and body weights of the animals of several groups. Both the positive control group and the second test group receiving 10 ml AP-01/kg body weight half an hour early showed an extremely significant difference compared to the negative control group; however, the other two test groups receiving 5 ml AP-01/kg and 10 ml AP-01/kg fed after ingestion of castor oil did not show any significant differences (*P* value <0.001). The gastric motility rate test using kaolin as a marker also showed a significant difference between the test groups and the negative control group. Here, the third test group receiving 10 ml AP-01/kg fed after half an hour of kaolin treatment showed a marked difference compared to the positive control group. In all three different measures of the percentage of inhibition, the positive control group and the second test group receiving 10 ml AP-01/kg body weight before receiving treatment showed a significant difference compared to the other two test groups (*P* value <0.01). Therefore, the statistical analysis of a multitude of factors revealed no significant difference among the positive control or the standard drug-receiving group and the second test group receiving 10 ml AP-01/kg body weight half an hour prior to receiving either castor oil or kaolin, meaning that both can produce equal and desired results in the decrease of diarrhea. However, the other two test groups did not show any extremely significant difference from the negative control group in all parameters. Hence, the comparable therapeutic efficacy could be achieved using a dose of 10 ml AP-01/kg body weight when given prior.

## 4. Discussion

Castor oil induces symptoms of diarrhea by releasing ricinoleic acid through the action of lipases in the gastric mucosa of mammalian animals. Ricinoleic acid triggers the release of prostaglandins following inflammatory activity produced on the upper gastrointestinal tract. Alteration in the permeability of gastric mucosal cells occurs with the release of prostaglandins, and a decrease in the absorption of electrolytes such as Na+ and K+ happens as a result. Due to the enhanced permeability and the hypersecretory effect of gastric mucosa, peristalsis hastens, and the symptoms of secretory diarrhea appear [2, 3]. Castor-oil-induced diarrhea typically has a predictable onset time after administration, allowing researchers to study the drug's onset of action and its ability to relieve diarrhea symptoms. It is a cost-effective and efficient method of screening potential antidiarrheal medications before moving on to more complex and expensive clinical trials. The ability to test drugs in a controlled laboratory setting using the castor oil-induced diarrhea model is a crucial step in the drug development pipeline [38, 39].

Formulations using *Holarrhena antidysenterica* stem bark as a major constituent were previously reported to be effective against secretory diarrhea. The presence of *Holarrhena antidysenterica* in the polyherbal AP-01 could be responsible for exerting antidiarrheal effects shown by the preparation. Prior studies suggest that the phytoconstituents such as alkaloids, tannins, and saponins have the effect of inhibiting intestinal motility and hydroelectrolytic secretions, which were mostly altered during diarrhea. The polyherbal preparation could act on the gastrointestinal stimulation by the activation of the histamine receptor and decrease the symptoms of diarrhea by relaxing the gastrointestinal tract by blocking Ca^2+^ ion channels and showing anticholinergic effects by inhibiting acetylcholinesterase locally. It eventually results in increasing the dry feces and decreasing the retention of the fecal material [40–43].

From the results of the current study, Ayurveda-based fermented polyherbal formulation AP-01 was found to be significantly effective against diarrhea induced by castor oil, suggesting that the polyherbal formulation could produce its action through the antisecretory mechanisms of action. The reduction of diarrhea in terms of the number of wet stools in the case of test groups was significant and comparable to the treatment effects of a standard drug used in positive control groups. The reduction of symptoms of diarrhea was found to be dose-dependent; however, there was a valuable finding that AP-01 could exert its antisecretory action even after introducing the diarrhea-causing substance. AP-01 not only delayed the onset of symptoms when administered prior but also reduced the time of incidence of appearance of symptoms and finally ceased any effects of castor oil-induced diarrhea.

Regularization of gastric motility signifies the restoration of normalcy after treatment with antidiarrheal and antisecretory agents. The longer duration of action of such drugs helps in increasing the dose intervals and stimulating patient compliance. The recent study shed some light on the antimotility effect of the polyherbal formulation AP-01. The assessment of in vivo passage of the drug was indirectly performed by using a marker (kaolin), which lightens the color of stool, which can be easily distinguished from the normal or wet stool color of rats [36], without affecting the pharmacodynamics. A significant hindrance to gastric motility was observed with AP-01 in comparison with the standard drugs where no significant effects were noticed after six to seven hours of administration. The efficacy of AP-01 in reducing gastric motility and the frequency of defecation was found to be most significant when an equal dose of AP-01 was administered one hour after in the case of the “third test group” in comparison to the “second test group,” suggesting some residual antimotility and antisecretory effects pertained by AP-01 as it might still be active on gastric mucosa before metabolism, excretion, and degradation of the formulation. The significant decrease in body weight and food intake in the case of the negative control group suggested that the symptoms of diarrhea disappeared, and the appetite and gastric conditions were yet to be recovered even after more than one day of treatment, whereas the animals of the other group were returned to normalcy. Further proof of this hypothesis was ascertained from the results showing a significant increase in the water uptake in the case of the negative control group, whereas other groups showed no significant change. It could be caused by excessive loss of water and electrolytes from the gastric mucosa of the animals fed with only castor oil, leaving them with a higher demand of water for the body to quench the loss. Animals belonging to all other groups showed no significant change in the water intake as the osmolarity and water balance in the gastrointestinal tract and the whole body, respectively, had become close to normal. The reduction in motility without significantly altering the amounts of food or water intake after treatment was shown with the administration of AP-01 at different dosages and administration times, which proved the formulation to be efficacious yet safe in the case of secretory diarrhea.

The Ayurveda-based “Arishtha” preparatory method was applied in formulating AP-01, which suggested boiling for a definite time would not affect the efficacy of the active constituents exerting pharmacological actions; therefore provided us with further scope in modification of dosage form and formulation and further detailed study of chemical and biochemical constituents that could lead us in demonstrating the exact mode of action of this polyherbal formulation, prepared by following Ayurveda in treating diarrhea and dysentery of various etiology. However, in vitro studies using individual plant components or unfermented polyherbal mixture showed no promising results compared to the actions exerted by the fermented polyherbal formulation AP-01 (data not shown).

Using human pathogenic bacteria for producing diarrhea in a murine model *per oral* route of administration was deemed unsuitable and unethical due to low body weight and possible consequences leading to animal sacrifices [33]. A zoological scale-up using either a canine model or rabbit model should have been adopted for future studies to develop a standard infectious diarrhea animal disease model using pathogenic bacteria, which was out of the scope of the current study. Therefore, in vitro bacterial susceptibility studies were conducted with AP-01 against all four GTPR strains, and the result showed the significant inhibition of the pathogenic bacteria with MICs within a feasible dosing regimen. The inhibition of swarming motility of *Salmonella* sp. by AP-01 at a sublethal dose was suggestive of the formulation being capable of hindering the wider spread of the virulent bacterial species within the affected epithelial mucosa, consequently minimizing the virulence and infection with time [44].

## 5. Conclusion

AP-01 was found efficacious against multiple antibiotic-resistant pathogens and safe for gastric mucosa, leaving an impression to research with the formulation with greater depth and detail. The mechanism of action could only be determined with further studies, including the detailed chemical analysis and structure-activity relationship determination so that this unused arsenal given by nature can be modified through cutting-edge technology in developing smarter dosage forms such as soft gel capsules, chewable pellets, or anything better suitable for better patient compliance and broader action spectrum.

Understanding the potential of fermented polyherbal formulations in treating conditions such as castor oil-induced diarrhea in animal models is not just about validating an alternative treatment. It touches on broader themes such as combating antibiotic resistance, advancing natural medicine, and improving gut health. This research could lead to significant advancements in both the prevention and treatment of gastrointestinal disorders, with far-reaching implications for public health and pharmaceutical innovation.

## Figures and Tables

**Table 1:** MTT assay-based cytotoxicity study of fermented polyherbal formulation.

Serial no	Contents	Percentage reduction in cell viability			Cytotoxicity
		RIE-1	IEC 18	HIEC 6	
1	DMSO	0	0	0	NA
2	10X MIC AP-01	7.92 ± 0.42	8.7 ± 0.64	8.33 ± 0.26	None
3	2X MIC AP-01	6.85 ± 0.18	7.22 ± 0.25	7.62 ± 0.72	None
4	MIC AP-01	5.79 ± 0.16	5.99 ± 0.19	5.11 ± 0.64	None
5	0.5 MIC AP-01	3.91 ± 0.27	3.77 ± 0.45	3.97 ± 0.15	None
6	0.25 MIC AP-01	2.56 ± 0.5	3.32 ± 0.41	2.83 ± 0.36	None

*P* < 0.05 expressed as mean ± standard deviation, an average of three determinations is represented in each value expressing mean ± standard deviation. *P* < 0.05 vs. control, Student's “*t*” test.

**Table 2:** Antibiotics resistance patterns based on CLSI/EUCAST zone of inhibition breakpoints.

Serial no	Antibiotic agent	Symbol	Disk content	CLSI breakpoint (resistance) mm or less	EUCAST breakpoint (resistance) mm or less	Experimental data *E. coli 01241* (mm)	Experimental data *S. typhi 01396* (mm)	Experimental data *S. typhi 01263* (mm)	Experimental data *S. boydii 01399* (mm)
1	Amikacin	AK	30 mcg	14	13	17	15	17	19
2	Amoxyclav	AMC	30 mcg	13	19	21	**11 [R]**	**18 [R]**	**13 [R]**
3	Ampicillin	AMP	10 mcg	13	14	**11 [R]**	15	19	**12 [R]**
4	Aztreonam	AT	30 mcg	17	21	**14 [R]**	22	23	22
5	Ceftazidime	CAZ	30 mcg	19	17	**12 [R]**	21	22	24
6	Ceftriaxone	CTR	30 mcg	19	20	**11 [R]**	22	24	23
7	Ciprofloxacin	CIP	5 mcg	20	20	23	**13 [R]**	**18 [R]**	**13 [R]**
8	Cefotaxime	CTX	30 mcg	22	NA	**12 [R]**	23	26	**14 [R]**
9	Doxycycline	DO	30 mcg	10	10	13	**9 [R]**	12	17
10	Erythromycin	E	15 mcg	13	16	18	**10 [R]**	19	20
11	Kanamycin	K	30 mcg	13	NA	16	24	17	**11 [R]**
12	Nalidixic acid	NA	30 mcg	13	12	**11 [R]**	17	**10 [R]**	**10 [R]**
13	Norfloxacin	NX	10 mcg	12	12	19	**9 [R]**	**9 [R]**	**9 [R]**
14	Ofloxacin	OF	5 mcg	NA	19	21	**15 [R]**	**17 [R]**	22
15	Streptomycin	S	10 mcg	11	NA	14	**8 [R]**	19	17
16	Trimethoprim/sulphamethoxazole	SXT	5 mcg	10	15	**8 [R]**	16	17	16
17	Cefepime	CPM	30 mcg	18	21	**13 [R]**	23	22	20
18	Doripenem	DOR	10 mcg	19	21	26	25	21	22
19	Imipenem	IPM	10 mcg	19	16	24	23	23	21
20	Meropenem	MRP	10 mcg	19	16	23	23	22	22

[R] designates resistance towards this antibiotic agent. Data point in bold signifies resistance which was double-checked.

**Table 3:** MIC and MBC of polyherbal AP-01 against bacterial strains of study.

Serial no	Bacterial strains	MIC *μ*l/ml	MBC *μ*l/ml
1	*Escherichia coli K12*	75	90
2	*Escherichia coli 01241*	80	120
3	*Salmonella typhi 01396*	100	150
4	*Salmonella typhi 01263*	100	150
5	*Shigella boydii 01399*	100	100

**Table 4:** Zone of inhibition shown by different volumes of AP-01 while keeping DMSO as negative control and meropenem as positive control.

Bacterial strains	Meropenem	AP-01 undiluted	AP-01 in DMSO (volume adjusted to 150 *µ*l)					DMSO
	10 mg	150 *µ*l	125 *µ*l	100 *µ*l	75 *µ*l	50 *µ*l	25 *µ*l	150 *µ*l
*Escherichia coli K12*	25.67 ± 0.58	25 ± 0.5	24.83 ± 0.58	25 ± 0.87	24.83 ± 0.29	16.75 ± 0.35	12.5 ± 0.5	0
*Escherichia coli 01241*	23.83 ± 0.29	22.67 ± 0.29	22.5 ± 0.5	22.33 ± 0.29	22.16 ± 0.29	15.67 ± 1.15	11.5 ± 0,5	0
*Salmonella typhi 01396*	23.67 ± 0.29	22.83 ± 0.58	22.67 ± 0.58	22.5 ± 0.5	16.17 ± 0.29	14.17 ± 0.76	13 ± 0.87	0
*Salmonella typhi 01263*	22.83 ± 0.76	22.33 ± 0.29	22.33 ± 0.29	21.83 ± 0.76	15.33 ± 0.58	15.17 ± 1.04	13.33 ± 0.58	0
*Shigella boydii 01399*	22.67 ± 0.58	21.17 ± 0.29	20.17 ± 0.76	20.17 ± 0.29	17.83 ± 0.76	15.67 ± 0.76	14.17 ± 0.29	0

*P* < 0.05 vs. control meropenem.

## Data Availability

The data that support the findings of this study are available within the article and also uploaded in the figure's files section.

## Abbreviations

AT: Aztreonam
AMC: Amoxiclav
AMP: Ampicillin
ATCC: American Type Culture Collection
CAZ: Ceftazidime
CIP: Ciprofloxacin
CTR: Ceftriaxone
CTX: Cefotaxime
CLSI: Clinical Laboratory Standards Institute
DEC: *Diarrheagenic Escherichia coli*
DMSO: Dimethyl sulphoxide
DO: Doxycycline
E: Erythromycin
EAEC: *Enteroaggregative E. Coli*
EHEC: *Enterohemorrhagic E. coli*
EIEC: *Enteroinvasive E. coli*
EPEC: *Enteropathogenic E. coli*
ETEC: *Enterotoxigenic E. coli*
EUCAST: European Committee on Antimicrobial Susceptibility Testing
FTIR: Fourier transform infrared spectroscopy
GTPR: Gastrointestinal Tract Pathogens Repository
HPLC-MS: High-performance liquid chromatography-mass spectrometry
ICMR: Indian Council of Medical Research
K: Kanamycin
MBC: Minimum bactericidal concentration
MIC: Minimum inhibitory concentration
MTT: (3-(4,5-Dimethylthiazol-2-yl)-2,5-diphenyltetrazolium bromide)
MW: Molecular weight
NA: Nalidixic acid
NICED: National Institute of Cholera and Enteric Diseases
NMR: Nuclear magnetic resonance
NOR: Norfloxacin
O: Ofloxacin
OD: Optical density
S: Streptomycin
SEM.: Standard error of mean
SXT: Trimethoprim/sulphamethoxazole
SD: Standard deviation.
